# Sleep Respiratory Disturbances in Girls with Rett Syndrome

**DOI:** 10.3390/ijerph192013082

**Published:** 2022-10-12

**Authors:** Xinyan Zhang, Marcel Smits, Leopold Curfs, Karen Spruyt

**Affiliations:** 1 NeuroDiderot-INSERM, Université de Paris, 75019 Paris, France; 2 Department of Sleep-Wake Disorders and Chronobiology, Hospital Gelderse Vallei Ede, Governor Kremers Centre, Maastricht University Medical Centre, 6716 RP Gelderland, The Netherlands; 3 Governor Kremers Centre, Maastricht University Medical Centre, 6200 MD Maastricht, The Netherlands

**Keywords:** *MECP2*, polysomnography, sleep-disordered breathing, Rett Syndrome

## Abstract

Individuals with Rett Syndrome (RTT), a rare neurodevelopmental disorder, present disordered breathing during wakefulness. Whilst findings on breathing during sleep are contradictory, the relation between sleep breathing and their clinical features, genetic characteristics, age, and sleep phase is rarely investigated, which is the objective of this study. Overnight polysomnography (PSG) was performed. Sleep macrostructure parameters were compared between the RTT subjects with and without sleep-disordered breathing (SDB). The association between the apnea–hypopnea index (AHI) with age at PSG was tested. Particularly for RTT subjects with SDB, the respiratory indexes in REM and NREM sleep were compared. Stratified analyses per clinical characteristics, genetic characteristics, and clinical features’ severity were performed. Non-parametric statistics were applied. A sample of 11 female RTT subjects, aged 8.69 ± 5.29 years with ten confirmed with *MECP2* mutations, were studied. The average AHI was 3.94 ± 1.19/h TST, of which eight (72.73%) had obstructive sleep apnea, i.e., six in 1/h TST ≤ AHI ≤ 5/h TST, and two in AHI > 5/h TST. The mean SpO_2_% was 81.00 ± 35.15%. The AHI was not significantly correlated with their age at PSG (r_s_ = −0.15, *p* = 0.67). Sleep macrostructure in SDB-absent and SDB-present groups was not different. Respiratory indexes in those with obstructive sleep apnea showed no difference between REM and NREM sleep nor any of the strata. In our clinical sample, more than half of the RTT subjects with *MECP2* mutations had obstructive sleep apnea in both NREM and REM sleep which was unrelated to their clinical features. Our results also indicated hypoxemia throughout nocturnal sleep in RTT. To conclude, our results suggest that disordered breathing during sleep is prevalently present in RTT as an independent clinical feature.

## 1. Introduction

Rett Syndrome (RTT, OMIM 312750) is a rare neurodevelopmental disorder with an incidence rate of 1/10,000 in girls [1]. Genetically, RTT is strongly associated with the X-linked dominant methyl-CpG-binding protein 2 (*MECP2*) gene [2], being confirmed in more than 90% of the RTT cases [3]. *MECP2* is a critical component regulating the DNA methylation and transcription of many other genes and is, therefore, expressed widely across the body systems. Thus, *MECP2* malfunction would cause diverse and complex consequences. In terms of symptomatology, RTT is characterized by stealthy onset in early infancy [4] and rapid developmental deterioration at approximately 6–18 months postnatal age [5]. A spectrum of cardinal clinical manifestations, including loss of acquired purposeful hand skills and spoken language and the presence of gait abnormalities and stereotypical hand movements [1], is generally seen in RTT. In addition to the essential clinical features, other features present in RTT are epilepsy, problematic sleeping, breathing dysfunction, and scoliosis. Upon presence and absence of these clinical features, individuals with RTT are diagnosed and classified into classic phenotype and atypical variants including three distinctive variants: Preserved speech variant (PSV), Congenital variant (CV), and Early seizure variant (ESV) [1].

The breathing disturbances of these patients are a predominant clinical abnormality during their wake state and are known to be potentially life-threatening [6]. More specifically, various breathing forms including apneustic breathing, episodic hyperventilation, air swallowing, forceful breathing, and breath-holding spells terminated by Valsalva maneuvers, have been vividly described [7,8,9,10]. Their daytime respiratory phenotypes were further classified into forceful, feeble, and apneustic [11], yet breathing disturbances are thought to vary between day and night [12]. Early studies reported roughly normal breathing patterns during sleep [13,14]. However, both obstructive sleep apnea (OSA) and central sleep apnea were reported in more recent RTT polysomnographic (PSG) studies [15,16,17]. Several sleep studies [15,16,17,18,19,20] reported that the apnea–hypopnea index (AHI), a key index to define sleep-disordered breathing (SDB), is in the abnormal range, thus suggestive of SDB in RTT. We further found in an aggregated sample from case (series) studies in the PSG literature [21], that 71.7% of RTT cases had an abnormal AHI, and approximately half of those with SDB were in the severe range (i.e., AHI >5/h TST). Additionally, we confirmed severe nocturnal hypoxemia with apneic events in RTT cases in our meta-analysis of PSG data [22].

In a typically developing (TD) population, the prevalence of SDB is estimated to vary widely from 0.7% to 13.0% [23]. Moreover, in the pediatric TD population, abnormal sleep breathing was found to be dominant [24,25,26] in rapid eye movement (REM) sleep. Findings of abnormal sleep breathing in specific sleep stages in RTT remain scant, such that only one study revealed a subtle decrease in oxygen saturation during REM sleep [13]. Furthermore, and firstly, in RTT animal studies, sleep apnea was found to be more frequent in non-rapid eye movement (NREM) sleep than in REM sleep in cyclin-dependent kinase-like 5 (i.e., *Cdkl5* associates to ESV variant) mutant mouse models [27]. Secondly, sleep macrostructure in RTT was found to be characterized by a poor REM-on switch resulting in abnormal sleep cycling [21]. Thirdly, previous studies [13,28] comparing sleep macrostructure in RTT with primary snoring girls showed no significant differences in sleep stage proportions. Altogether, this questions the issue of sleep breathing and its potential role in the sleep macrostructure of RTT.

In addition, we found the salient tendency of sleep macrostructure perturbations varying along with several cardinal clinical features, particularly regarding hand functioning in our previous study (under revision), yet the association between sleep breathing abnormalities and these clinical features has been unexplored previously.

Therefore, in the present study, we performed overnight PSG recordings in RTT girls with *MECP2* mutations to characterize sleep breathing abnormalities in RTT, to examine the hypotheses that (1) sleep breathing abnormalities in the individuals with RTT are present since early childhood, (2) sleep macrostructure between RTT girls with and without SDB is different, (3) the sleep breathing disturbances differ during REM and NREM sleep, and (4) the severity of SDB may aggravate with the cardinal clinical features’ severity in RTT. Consequently, we aimed to delineate the characteristics of sleep breathing abnormalities and their association with other core clinical and genetic features in individuals with RTT, especially regarding individuals with *MECP2* mutations.

## 2. Materials and Methods

### 2.1. RTT Participants and Sleep Recording

RTT subjects confirmed by diagnostic criteria [1] and genetic tests were invited for sleep recording and assessment of clinical features’ severity from 2013 to 2016, following the institutional guidelines and the 1964 Declaration of Helsinki. Informed consent was obtained from parents for use of their anonymized data, given that the study was approved by the IRB (IRB 2022-3158). RTT subjects with other central nervous system complications would be excluded.

PSG recording was performed and scored according to the AASM 2007 guideline [29]. Standard sleep macrostructure parameters are reported in this study; they are total sleep time (i.e., TST, the time from sleep onset to the end of the final sleep epoch minus wake after sleep onset), sleep onset latency (i.e., SOL, time from lights-off to sleep onset), wake after sleep onset (i.e., WASO, the proportion of time spent awake during the time in bed, expressed as a percentage), and proportional parameters including sleep efficiency (i.e., SEI, the ratio between TST and time from lights-off in the evening to lights-on in the next morning, expressed as a percentage), stage N (i.e., the amount of time in NREM stages per TST, expressed as a percentage, being stage N1, stage N2, and stage N3), and stage R (i.e., the amount of time in REM sleep per TST, expressed as a percentage). 

For sleep breathing, respiratory-event-related parameters were scored per hour of TST, NREM sleep, and REM sleep, respectively, including AHI (i.e., normal value: ≤1/h TST, with >5/h TST further defined as severe SDB), apnea index, and hypopnea index. O_2_-saturation-related parameters were calculated only for TST, including mean SpO_2_%, nadir SpO_2_% (i.e., normal value: >90%), and oxygen desaturation index (i.e., ODI, drop 3% or 4% below baseline O_2_ levels).

### 2.2. Experimental Design and According to RTT Group Stratification

The data of AHI in all RTT participants were used to analyze the association between sleep breathing abnormalities and age. Next, RTT subjects were divided into subgroups of those with and without SDB (i.e., AHI > 1/h TST as presenting SDB), for comparing their sleep macrostructure. 

Then, to investigate the sleep breathing abnormalities in REM and NREM sleep, we compared the key sleep breathing parameters of the SDB-present subgroup. 

Lastly, to analyze the differences in sleep breathing abnormalities upon other RTT core clinical and genetic characteristics, we also stratified only the SDB-present subjects per clinical characteristics, genetic characteristics, and severity levels of clinical features. In clinical characteristics, phenotype (e.g., FF: forme frust variant and PSV: preserved speech variant), stage (e.g., I: Early-Onset Stagnation Period, II: Rapid Developmental Regression Period, III: Pseudo-Stationary Stage, and IV: Late Motor Deterioration, including IVA and IVB), and breathing phenotype (i.e., feeble, forceful, and other) were present. For the genetic characteristics, mutation domain (e.g., Methyl-CpG-Binding Domain (MBD), Transcription Repression Domain (TRD), and C-Terminal Segment (CTS)) and mutation type (e.g., nonsense mutation (ns) and missense mutation (ms)) were present. For the severity levels of clinical features, a three-level category (i.e., mild, moderate, and severe) was present in five clinical features of hand function, sitting, walking, speech, and scoliosis, and a two-level category (i.e., present and absent) was applied to epilepsy. 

### 2.3. Statistical Analysis

Descriptive results (mean ± standard deviation) are reported per presence of SDB, in REM and NREM sleep as well as per stratification of genetic characteristics or clinical features. Spearman’s correlation analysis was applied to examine the correlation between AHI and chronological age at PSG. Cohen’s *d* was applied for calculating the standardized mean differences, facilitating the clinical interpretation of the data (small: *d* < 0.2; 0.2 > medium < 0.5, and >0.5 large). Mann–Whitney *U* test was performed to compare the sleep macrostructure parameters between SDB-absent and SDB-present groups. Sign tests were used to compare the apneic indexes between stage NREM and stage REM in the SDB group. Kruskal–Wallis *H* test was used to compare sleep macrostructure parameters in different strata. Statistical analyses were performed in Statistica version 13 (TIBCO Software Inc. (2018), Palo Alto, CA, USA). A *p*-value was set at 0.05 as statistical significance.

## 3. Results

### 3.1. Sample Description

The 11 RTT subjects (aged 8.69 ± 5.29 years) were primarily classic phenotype (i.e., *n* = 7), with one PSV variant and one FF variant. Six were in stage III or stage IV. For the genetic mutations, all 11 subjects but one were known to have MECP2 mutations. Regarding specific nucleotide changes, two were of 502C > T and one RTT subject was in each of the following: c. 808C, gross rear, 806delG, 816C > T, 897C > T, c.880C > T, 880C > T, and 951_968 delinsAG. Regarding the mutation type and mutation domain, there were two subjects of ‘ms’ and seven of ‘ns’, four in MBD, four in TRD, and one in CTS. 

The average AHI of the 11 RTT subjects was 3.94 ± 1.19/h TST, of which 27.3% had AHI < 1/h TST, 54.5% in 1/h TST ≤ AHI ≤ 5/h TST, and 18.2% in AHI > 5/h TST. We found no significant correlation between their AHI and age at PSG (r_s_ = −0.15, *p* = 0.67).

### 3.2. Sleep Macrostructure upon the Presence of SDB

Based on the AHI > 1/h TST, eight (72.73%) RTT subjects had sleep apnea. Sleep macrostructure of SDB-absent (*n* = 3) and SDB-present (*n* = 8) groups is shown in Table 1. Cohen’s *d* of sleep macrostructure parameters between SDB-absent and SDB-present subgroups were mostly large, and we found no significant difference in their sleep macrostructure parameters.

### 3.3. Sleep-Disordered Breathing in the REM and NREM Sleep

The results of sleep respiratory parameters in those with SDB (*n* = 8) were tabulated and are shown in Table 2 for TST, REM, and NREM sleep, respectively. The oxygen-saturation-related indexes were available only for TST (*n* = 7); the mean SpO_2_% was 81.00 ± 35.15%, nadir SpO_2_% was 85.43 ± 6.88%, and ODI was 20.40 ± 31.33/h TST. Whereas the standardized mean differences of apneic indexes between REM and NREM sleep were of medium size, the sign test showed no significant difference (presented in Table 2).

### 3.4. Sleep-Disordered Breathing upon the Genetic Characteristics and Clinical Features’ Severity

We found no significant difference in the comparison of sleep respiratory indexes among the strata per genetic and clinical phenotypic characteristics nor severity levels of clinical features, as shown in Table 3. The standardized mean difference is summarized in Supplementary Table S1. Given the Cohen’s *d* values, several clinical differences are noticeable. Upon genetic characteristics, per mutation type, the individuals with ms mutation type may have more apneic events but higher saturation, which is the opposite for individuals with the ns mutation type. Nadir SpO_2_ (%) in individuals with a mutation in the TRD domain was the lowest. Upon the clinical items’ severity, individuals without epilepsy and sitting abnormalities showed clinically more apneic events. Individuals with motor issues such as non-functional hand use, walking with support, or unable to walk demonstrated saturation problems. 

## 4. Discussion

This is the first study in a homogeneous RTT group with *MECP2* mutations to analyze SDB in relation to their clinical and genetic characteristics. We found in our sample that more than half of the girls with *MECP2* mutations presented sleep apnea. Further, sleep macrostructure appeared to be unaffected by the presence of sleep apnea. Although the severity of the apneic events tended to be greater in REM sleep than in NREM sleep, there was no significant mean difference between the sleep phases. Our analysis also revealed that SBD was constant irrespective of RTT clinical phenotypes, such as disease stages, genetic characteristics, and the severity of their cardinal features. Therefore, our results suggest that disordered breathing during sleep is prevalently present in RTT as an independent clinical feature. Within the realm of a sample with only *MECP2* mutations, our findings are insufficient to explain its mechanism, yet the genetic mutation might be the mainstream regulator of the sleep-breathing pathophysiology.

### 4.1. Sleep Breathing in RTT

Although their breathing during sleep seems to show fewer abnormalities compared with their disturbed breathing during wakefulness, we confirmed SDB in the majority of our RTT sample cases. Previously, one study reported comparable sleep breathing to healthy subjects in RTT [14], whilst another early study concluded roughly normal breathing during sleep compared with primary snoring subjects [13]. The latter study noted only subtle decreases in oxygen saturation during REM sleep in their RTT sample, which may demonstrate the increased upper airway resistance during sleep in RTT comparable to primary snoring [30]. More recent studies have reported sleep breathing abnormalities in their RTT samples such as central sleep apnea and hypoxemia [15,17]. Overall, a prevalence rate of SDB (i.e., by AHI >1/h TST) in RTT of greater than 70% was described. Upon reviewing the sample characteristics across these studies, we have to note that in the early study by Glaze et al. [14], RTT subjects were diagnosed only by fulfilling the clinical RTT features, and the recent studies mostly confirmed RTT with genetic mutations, as in our study. Furthermore, our RTT sample involves only girls with *MECP2* mutations. Thus, possibly upon a confirmed genetic mutation, and hence a shared pathological basis, the potential bias due to the sample characteristics as seen in other studies might be less confounding in ours. 

In terms of the pathophysiological mechanism behind the sleep-breathing abnormalities in RTT, firstly, only a few animal studies focused on genetic mutations such as *Mecp2* [31] and *Cdkl5* [27,32]. Mouse models of both genetic mutations presented apnea during sleep. That is, more sleep apneas with increasing age in *Mecp2* mutant mice [31] and more frequently during NREM sleep in *Cdkl5* mutant ones [27] have been reported. In our study, including only girls with *MECP2* mutations, we found a high prevalence of SDB. However, our female sample with *MECP2* mutations did not show a significant correlation with their chronological age, which might be due to the phenotypic variances caused by differences from sex-determining genes and genetic alterations. That is, in the animal model the type was *Mecp2*-null (i.e., *Mecp2*^-/y^), and in human subjects, nucleotide changes were mutant fragment(s) in the *MECP2* gene.

Secondly, the control of coordinated ventilation involves the integration of chemoreceptor input, motor input, as well as behavioral input. During sleep, predominantly chemoreception is involved, whereas in the waking state, it is the behavioral regulation. Given that disturbed breathing during sleep was present in our sample with *MECP2* mutation, and also reported in previous studies [15,16,21], such a finding may imply that the intermitted breathing regulation is elicited by hypercapnia or metabolic acidosis. Further, such chemical feedback circuits may be passivated due to decreased sensitivity of chemoreceptors under long-term hypoxemia [33]. More studies suggested that abnormal sleep breathing in RTT is associated with poor ventilatory control given the brainstem dysfunction [11,34], yet the specific pathophysiological pathways behind SDB in RTT are still uncertain. 

Thirdly, SDB is primarily attributed to a narrowing upper airway during sleep, which is usually associated with the following possible causes: (1) a (temporary) inability of the upper airway dilating muscles to withstand the negative forces generated within the upper airway during inspiration, (2) abnormal craniofacial anatomy, soft tissue accumulation in the oropharynx, and (3) increasing collapsing forces of airway for rostral fluid [35]. In RTT the weakened throat anatomy presenting as dysphagia, e.g., feeding difficulties, oral apraxia, and dyskinetic tongue movements [36], is acknowledged. However, we found no significant difference in sleep breathing parameters upon a history of adeno(tonsil)ectomy surgery (A&T) in an aggregated literature RTT sample [21].

### 4.2. Sleep Macrostructure upon the Presence of SDB in RTT

We could not confirm our hypothesis that sleep macrostructure between RTT girls with and without SDB is different, since in this study the sleep macrostructure in girls with RTT did not differ upon the presence of SDB. However, based on Cohen’s *d*—although all are large effect sizes—particularly SEI (hence higher WASO) and Stage N3 were lower in individuals suffering from SDB. Firstly, it is known that an abnormal AHI indicates the presence of airway obstruction during sleep, which is usually terminated by brief (micro-) arousals, causing sleep fragmentation [37]. Additionally, in RTT subjects [38,39,40] night waking is prevalent and in RTT animal sleep studies [41] fragmented sleep was summarized. Yet, in our study, the arousal index was similar across groups, irrespective of the presence of SDB. We have to note here, however, that in infants and younger children, the majority of respiratory events are found not to be terminated with spontaneous arousals, irrespective of quiet (or NREM) and active (REM) sleep [42]. Secondly, for other possible sleep macrostructure perturbations related to abnormal sleep breathing, in the studies comparing sleep stage distribution between children with OSA and healthy ones, researchers reported increased stage N1 and decreased stage N3 in the OSA group [43], whilst others reported non-significant differences [44,45]. In this study, for an RTT sample, we found similar stage proportions irrespective of the presence of SDB: yet, clinically, a poorer quality of sleep might be suspected (Cohen’s *d* findings). Furthermore, in our previous review of aggregated literature on RTT cases and comparing their sleep with TD children, we found decreased stage N1 and increased stage N3 in RTT cases with *MECP2* mutations. In fact, the sample also included those with AHI > 1/h TST [21]. In other words, the sleep stage distribution changes in individuals with RTT did not mimic the sleep macrostructure findings as reported in OSA samples. None of our subjects demonstrated central sleep apnea. In children with idiopathic central sleep apnea (ICSA), significantly higher stage N2 and lower stage N3 [46] have been reported, which was also not found in our sample. Consequently, not the disordered breathing during sleep but potentially the genetic mutations override the perturbations in the sleep macrostructure of individuals with RTT.

### 4.3. Sleep Breathing Abnormalities and Sleep Phases in RTT

In TD children, SDB was found to be predominately present in REM sleep [26,47,48], and in adults, it occurred more frequently during deeper sleep stages whilst being more severe in REM sleep [49,50]. For children with OSA, one study reported that the obstructive events occur throughout REM (55%) and NREM (45%) phases but without a difference in desaturation episodes in REM and NREM sleep [44]. This again confirms the susceptibility of REM sleep toward apneic events. Contrary, we found that the apneic indexes were not different between REM and NREM sleep in RTT subjects with SDB, but clinically they showed a tendency to be frequent in REM sleep (Cohen’s *d*). We, thus, could not confirm our hypothesis regarding the sleep phase differences in sleep breathing abnormalities for a homogeneous RTT sample. It could be that there is a negative publication bias because thus far only one study explored the issue of sleep phases. Namely, Marcus et al. [13] reported a subtle decreased oxygen saturation during REM when comparing RTT cases with primary snoring subjects [13]. Additionally, we have to note that REM sleep is proven to be attenuated in RTT [20,21]. Linking to the potential regulation pathways [51] during sleep, such even distribution of sleep apneic events may indicate an impaired breathing generation in RTT. In *Mecp2*-null male mice (during wakefulness), a deficiency in noradrenergic and serotonergic modulation of the medullary respiratory network was suspected to be related to their worsening of breathing abnormalities with age [52]. However, such pathways have not yet been specified for the sleep phase.

### 4.4. Sleep Breathing Abnormalities and Characteristics in RTT Subjects

The stratification analysis in our study indicated that SDB in the RTT sample with *MECP2* mutations did not vary with the clinical characteristics: again, we could not confirm our hypothesis. Contrary to our previous findings on sleep macrostructure (under revision), the presence of sleep apnea events seems not to be affected by the severity of psychomotor dysfunctioning. Our negative findings could be explained in several ways. On the one hand, we only included RTT subjects with *MECP2* mutations, potentially sharing common fundamental pathologic molecular pathways as well as clinical features. On the other hand, as mentioned, ventilatory regulation differs significantly between sleep and wakefulness. With sleep onset, behavioral-related inputting signals terminate, such that the voluntary drive to both the respiratory pump muscles and the upper airway dilating muscles is lost. Further, the usual ventilatory responses to hypoxemic and hypercapnic levels also decrease, especially during REM sleep [51]. Such a physiological basis is important for the pathogenesis of upper airway obstruction during sleep. Additionally, the metabolic responses to fluctuations in oxygen and carbon dioxide saturation dominate the respiratory impulse during sleep. Regarding the clinical features within our analysis (e.g., breathing type), brainstem dysfunction may also provide influence, but per a different downstream mechanism. Lastly, and contrary to others, we did not use a healthy or snoring comparison group but, rather, RTT subjects. 

Thus, on the basis of the RTT strata results, integrating that *MECP2* was found to be required for an appropriate post-inspiratory response rate from acute hypoxia [33,53] during wakefulness but not directly damage the basic respiratory rhythm generation [52], we may conclude that despite the acknowledged brainstem impairments, the baseline sympathetic tone is preserved in RTT [7]. That is, though breathing rhythm and the response to episodes of hypoxia, hypercapnia, and obstructive apnea might be affected by *MECP2* mutations, their respiratory system is still able to centrally generate a normal respiratory rhythm during sleep independently. 

Clinically, based on Cohen’s *d*, individuals with TRD of MECP2 showed lower nadir saturation compared with individuals with MBD of MECP2, which warrants further investigation. In addition, individuals with motor issues such as non-functional hand use, walking with support, or being unable to walk demonstrated saturation problems. 

Regarding the potential neurotransmitters, disturbed monoaminergic modulation including noradrenaline (NA) and serotonin (5-HT), which are involved in both sleep-wake cycles [54] and breathing regulation [55], have been related to the sleep-breathing abnormalities in RTT [56]. However, only reported in *Mecp2*-null mice, the brain levels of NA and 5-HT are normal at birth and then decline from 14 postnatal days (i.e., represents approximately 2 years of age in humans [57]) onwards [58]. 

### 4.5. Limitations

Constraints exist in this study. That is, only girls with *MECP2* mutations were involved in this study; thus, discussion on and generalizability to *CDKL5* and Forkhead box G1 (*FOXG1*) sleep breathing is not possible. The unequal sample size may lead to a loss of statistical power by imbalanced variances between samples, yet non-parametric tests were applied. Small samples are inherent to research on rare disorders. An aggregated analysis of cases from the literature suggested 71.7% of the cases demonstrated SDB; therefore, our sample with 8 out of 11(72.7%) consequently had sufficient power.

## 5. Conclusions

In conclusion, we found that a portion of the RTT subjects with *MECP2* mutations presented apneic events in both NREM and REM sleep, with hypoxemia throughout the nocturnal phase, which was unrelated to their clinical features.

## Figures and Tables

**Table 1:** Sleep macrostructure of RTT girls with and without sleep-disordered breathing.

Parameters	SDB-Absent RTT (*n* = 3)	SDB-Present RTT (*n* = 8)	The Standardized Mean Difference between SDB-Absent and SDB-Present	Mann–Whitney Test between SDB-Absent and SDB-Present	
	Mean ± SD	Mean ± SD	Cohen’s *d*	*U*	*p*-Value
TST (min)	614.67 ± 80.12	529.75 ± 105.29	0.85	4.00	0.13
SEI (%)	92.20 ± 2.51	77.29 ± 12.65	1.33	2.00	*0.05*
SOL (min)	20.10 ± 9.90	20.33 ± 15.47	−0.02	12.00	0.92
WASO (%)	7.80 ± 2.51	22.71 ± 12.65	−1.33	2.00	*0.05*
Stage N1 (%)	4.53 ± 3.47	8.29 ± 6.19	−0.66	8.00	0.47
Stage N2 (%)	27.03 ± 6.95	36.55 ± 12.12	−0.85	5.00	0.18
Stage N3 (%)	50.30 ± 5.28	42.36 ± 9.53	0.91	6.00	0.26
Stage R (%)	18.13 ± 4.04	12.79 ± 8.28	0.71	8.00	0.47
Arousal index *	27.30	7.78 ± 3.59	-	-	-

*: Only one RTT subject of the SDB-absent group and five in the SDB-present group showed arousals. RTT: Rett Syndrome; SD: standard deviation; SDB: sleep-disordered breathing; SEI: sleep efficiency; SOL: sleep onset latency; Stage N: the amount of time in non-rapid eye movement sleep stages per TST, expressed as a percentage, including stage N1, stage N2, and stage N3; Stage R: the amount of time in rapid eye movement sleep per TST, expressed as a percentage; TST: total sleep time; and WASO: wake after sleep onset.

**Table 2:** The comparison between REM and NREM sleep of the sleep respiratory indexes in RTT subjects (*n* = 8) with sleep-disordered breathing.

Parameters	TST		REM Sleep		NREM Sleep		The Standardized Mean Difference between REM and NREM Sleep	Sign Test between REM and NREM Sleep	
	*n*	Mean ± SD	*n*	Mean ± SD	*n*	Mean ± SD	Cohen’s *d*	Z-Value	*p*-Value
AHI	8	5.31 ± 4.00	8	6.83 ± 5.08	8	4.93 ± 4.15	−0.41	0	1
Apnea index	7	3.27 ± 3.56	6	4.52 ± 3.54	7	3.04 ± 3.71	−0.41	1.22	0.22
Hypopnea index	8	2.43 ± 1.81	7	3.90 ± 4.35	8	2.26 ± 1.76	−0.54	0.95	0.34
Respiratory-related MA	4	1.35 ± 0.97	1	1.00	4	1.50 ± 1.12	-	1.50	0.13

AHI: apnea–hypopnea index; MA: micro arousal; NREM: non-rapid eye movement sleep; REM: rapid eye movement; TST: total sleep time; and SD: standard deviation.

**Table 3:** Difference of sleep respiratory parameters in RTT clinical and genetic stratifications in the SDB-present RTT subjects (*n* = 8).

RTT Group [*n*]	Stratum	AHI Mean ± SD [*n*]	Apnea Index Mean ± SD [*n*]	Hypopnea Index Mean ± SD [*n*]	ODI Mean ± SD [*n*]	Mean SpO_2_ (%) Mean ± SD [*n*]	Nadir SpO_2_ (%) Mean ± SD [*n*]
**SDB-present [8]**	SDB-present	5.31 ± 4.00 [8]	3.27 ± 3.56 [7]	2.43 ± 1.81 [7]	20.40 ±31.33 [7]	81.00 ± 35.15 [7]	85.43 ± 6.88 [7]
**Stratification per Genetic characteristic**							
**Mutation type**	ms	7.70 ± 7.35 [2]	6.00 ± 6.93 [2]	1.70 ± 0.42 [2]	8.00 ± 7.35 [2]	97.50 ± 0.71 [2]	90.00 ± 1.41 [2]
	ns	4.18 ± 2.15 [4]	2.73 ± 1.27 [3]	2.10 ± 1.00 [4]	26.88 ± 42.26 [4]	71.50 ± 46.48 [4]	86.00 ± 6.00[4]
	Missing	5.20 ± 5.23 [2]	1.35 ± 1.34 [2]	3.80 ± 3.82 [2]	19.30 [1]	86.00 [1]	74.00 [1]
K–W test within group	H [*n*] df = 1, *p*-value	1.38 [6], 0.24	0 [5], 1.00	0.21 [6], 0.64	0 [6], 1.00	0.86 [6], 0.35	0.24 [6], 0.62
**Mutation domain**	CTS	1.10 [1]	[0]	1.10 [1]	3.70 [1]	95.00 [1]	91.00 [1]
	MBD	6.57 ± 5.56 [3]	4.67 ± 5.42 [3]	1.90 ± 0.46 [3]	6.17 ± 6.09 [3]	98.00 [3]	90.33 ± 1.15 [3]
	TRD	5.65 ± 0.21 [2]	3.10 ± 1.56 [2]	2.50 ± 1.27 [2]	50.65 ± 55.65 [2]	46.00 ± 62.23 [2]	81.00 ± 2.83 [2]
	Missing	5.20 ± 5.23 [2]	1.35 ± 1.34 [2]	3.80 ± 3.82 [2]	19.30 [1]	86.00 [1]	74.00 [1]
K–W test within group	H [*n*] df = 2, *p*-value	2.38 [6], 0.30	0 [5], 1.00	2.38 [6], 0.30	1.95 [6], 0.38	4.29 [6], 0.12	3.98 [6], 0.14
**Stratification per** **Clinical characteristic**							
**RTT phenotype**	CR	5.40 ± 2.35 [5]	2.32 ± 1.14 [5]	3.04 ± 2.09 [5]	25.18 ± 36.89 [5]	75.00 ± 41.17 [5]	83.60 ± 7.47 [5]
	FF	1.10 [1]	[0]	1.10 [1]	3.70 [1]	95.00 [1]	91.00 [1]
	PSV	12.90 [1]	10.90 [1]	2.00 [1]	13.20 [1]	97.00 [1]	89.00 [1]
	Missing	1.50 [1]	0.40 [1]	1.10 [1]	[0]	[0]	[0]
K–W test within group	H [*n*] df = 2, *p*-value	3.86 [7], 0.15	0 [6], 1.00	2.31 [7], 0.31	0.43 [7], 0.81	0.26 [7], 0.88	1.11 [7], 0.57
**RTT stage**	II	8.90 [1]	2.30 [1]	6.50 [1]	19.30 [1]	86.00 [1]	74.00 [1]
	III	3.40 ± 1.27 [2]	1.55 ± 0.64 [2]	1.85 ± 0.64 [2]	2.65 ± 0.21 [2]	98.50 ± 0.71 [2]	91.00 ± 0.00 [2]
	III-IVA	5.50 [1]	2.00 [1]	3.40 [1]	11.30 [1]	2.00 [1]	83.00 [1]
	IVB	5.80 [1]	4.20 [1]	1.60 [1]	90.00 [1]	90.00 [1]	79.00 [1]
	Missing	5.17 ± 6.70 [3]	5.65 ± 7.42 [2]	1.40 ± 0.52 [3]	8.45 ± 6.71 [2]	96.00 ± 1.41 [2]	90.00 ± 1.41 [2]
K–W test within group	H [*n*] df = 3, *p*-value	3.80 [5], 0.28	3.53 [5], 0.32	3.20 [5], 0.36	3.80 [5], 0.28	3.80 [5], 0.28	4.00 [5], 0.26
**Breathing type**	Forceful	3.17 ± 2.08 [3]	1.67 ± 0.80 [3]	1.97 ± 1.25 [3]	7.05 ± 6.01 [2]	50.00 ± 67.88 [2]	87.00 ± 5.66 [2]
	Feeble	5.80 [1]	4.20 [1]	1.60 [1]	90.00 [1]	90.00 [1]	79.00 [1]
	Other	4.30 [1]	2.00 [1]	2.30 [1]	2.50 [1]	99.00 [1]	91.00 [1]
	Missing	7.63 ± 6.00 [3]	6.60 ± 6.08 [2]	3.20 ± 2.89 [3]	12.07 ± 7.86 [3]	92.67 ± 5.86 [3]	84.67 ± 9.29 [3]
K–W test within group	H [*n*] df = 2, *p*-value	2.13 [5], 0.34	2.67 [5], 0.26	0.53 [5], 0.77	2.70 [4], 0.26	1.80 [4], 0.41	2.25 [4], 0.32
**Stratification per** **Clinical severity item**							
**Epilepsy**	Not have	8.10 ± 5.25 [3]	4.77 ± 5.35 [3]	3.30 ± 2.88 [3]	11.77 ± 8.34 [3]	93.67 ± 6.66 [3]	84.67 ± 9.29 [3]
	Have	4.18 ± 2.15 [4]	2.73 ± 1.27[3]	2.10 ± 1.00 [4]	26.88 ± 42.26 [4]	71.50 ± 46.48 [4]	86.00 ± 6.00 [4]
	Missing	1.50 [1]	0.40 [1]	1.10 [1]	[0]	[0]	[0]
K–W test within group	H [*n*] df = 1, *p*-value	1.13 [7], 0.29	0.05 [6], 0.82	0.13 [7], 0.72	0.13 [7], 0.72	0.13 [7], 0.72	0.13 [7], 0.71
**Hand function**	No hand functional abnormality	7.00 ± 8.34 [2]	10.90 [1]	1.55 ± 0.64 [2]	8.45 ± 6.72 [2]	96.00 ± 1.41 [2]	90.00 ± 1.41 [2]
	Reduced/poor hand use	4.30 [1]	2.00 [1]	2.30 [1]	2.50 [1]	99.00 [1]	91.00 [1]
	Non-functional hand use	5.68 ± 2.62 [4]	2.40 ± 1.30 [4]	3.23 ± 2.36 [4]	30.85 ± 40.00 [4]	69.00 ± 44.94 [4]	81.75 ± 7.18 [4]
	Missing	1.50 [1]	0.40 [1]	1.10 [1]	[0]	[0]	[0]
K–W test within group	H [*n*] df = 2, *p*-value	0.27 [7], 0.87	2.30 [6], 0.32	1.39 [7], 0.50	2.41 [7], 0.30	2.89 [7], 0.24	2.31 [7], 0.32
**Sitting**	No sitting abnormality	6.50 ± 5.96 [3]	6.45 ± 6.29 [2]	2.17 ± 1.16 [3]	9.40 ± 5.03 [3]	64.67 ± 54.28 [3]	87.67 ± 4.16 [3]
	Sitting with support	4.30 [1]	2.00 [1]	2.30 [1]	2.50 [1]	99.00 [1]	91.00 [1]
	Unable	5.73 ± 3.20 [3]	2.53 ± 1.56 [3]	3.17 ± 2.89 [3]	37.37 ± 46.32 [3]	91.33 ± 6.11 [3]	81.33 ± 8.74 [3]
	Missing	1.50 [1]	0.40 [1]	1.10 [1]	[0]	[0]	[0]
K–W test within group	H [*n*] df = 2, *p*-value	0.29 [7], 0.87	0.65 [6], 0.72	0.29 [7], 0.87	2.57 [7], 0.28	2.29 [7], 0.32	1.69 [7], 0.43
**Walking**	No walking abnormality	7.00 ± 8.34 [2]	10.90 [1]	1.55 ± 0.64 [2]	8.45 ± 6.72 [2]	96.00 ± 1.41 [2]	90.00 ± 1.41 [2]
	Walking with support	4.90 ± 0.85 [2]	2.00 ± 0.00 [2]	2.85 ± 0.78 [2]	6.90 ± 6.22 [2]	50.50 ± 68.59 [2]	87.00 ± 5.66 [2]
	Unable	5.73 ± 3.20 [3]	2.53 ± 1.56 [3]	3.17 ± 2.89 [3]	37.37 ± 46.32 [3]	91.33 ± 6.11 [3]	81.33 ± 8.74 [3]
	Missing	1.50 [1]	0.40 [1]	1.10 [1]	[0]	[0]	[0]
K–W test within group	H [*n*] df = 2, *p*-value	0.18 [7], 0.91	2.45 [6], 0.29	1.93 [7], 0.38	1.61 [7], 0.45	0.18 [7], 0.91	1.27 [7], 0.53
**Spoken language**	No spoken abnormality	1.10 [1]	[0]	1.10 [1]	3.70 [1]	95.00 [1]	91.00 [1]
	Use some real words with meaning	12.90 [1]	10.90 [1]	2.00 [1]	13.20 [1]	97.00 [1]	89.00 [1]
	Use no real words with meaning	5.40 ± 2.35 [5]	2.32 ± 1.14 [5]	3.04 ± 2.09 [5]	25.18 ± 36.89 [5]	75.00 ± 41.17 [5]	83.60 ± 7.47 [5]
	Missing	1.50 [1]	0.40 [1]	1.10 [1]	[0]	[0]	[0]
K–W test within group	H [*n*] df = 1, *p*-value	3.86 [7], 0.15	0 [6], 1.00	2.31 [7], 0.31	0.43 [7], 0.81	0.26 [7], 0.88	1.11 [7], 0.57
**Scoliosis**	Had no deviation	6.58 ± 5.26 [4]	4.77 ± 5.35 [3]	2.98 ± 2.37 [4]	9.75 ± 7.92 [4]	94.00 ± 5.48 [4]	86.25 ± 8.22 [4]
	Mild scoliosis	4.30 [1]	1.80 [1]	2.3 [1]	2.5 [1]	99.00 [1]	91.00 [1]
	Severe scoliosis	5.65 ± 0.21 [2]	3.10 ± 1.56 [2]	2.50 ± 1.27 [2]	50.65 ± 55.65 [2]	46.00 ± 62.23 [2]	81.00 ± 2.23 [2]
	Missing	1.50 [1]	0.40 [1]	1.10 [1]	[0]	[0]	[0]
K–W test within group	H [*n*] df = 1, *p*-value	0.32 [7], 0.85	0.35 [6], 0.84	0.54 [7], 0.77	2.89 [7], 0.24	3.70 [7], 0.16	2.02 [7], 0.36

AHI: apnea–hypopnea index; CR: classic RTT phenotype; CTS: C-Terminal Segment; FF: forme frust variant; H: Kruskal–Wallis test; II: Rapid Developmental Regression Period; III: Pseudo-Stationary Stage; IV: Late Motor Deterioration; MBD: Methyl-CpG-Binding Domain; ms: missense mutation; ns: nonsense mutation; ODI: oxygen desaturation index; PSV: preserved speech variant; RTT: Rett Syndrome; SD: standard deviation; SDB: sleep-disordered breathing; and TRD: Transcription Repression Domain.

## Data Availability

Not applicable.

## Supplementary Materials

The following supporting information can be downloaded at: https://www.mdpi.com/article/10.3390/ijerph192013082/s1, Table S1: Cohen’s *d* of RTT strata.
