# Supplementary material for: Sleep Respiratory Disturbances in Girls with Rett Syndrome
Source: Int J Environ Res Public Health. 2022 Oct 12;19(20):13082. doi: 10.3390/ijerph192013082 (PMC9602589; doi:10.3390/ijerph192013082)
Supplement: Supplementary file 1 [file ijerph-19-13082-s001.zip › ijerph-1925259-supplementary.pdf]

International Journal of
Environmental Research and Public Health

MDPI

Supplementary Table S1. Cohen's *d* of RTT strata.

Stratification		RTT stratum	Statistics	AHI	Apnea Index	Hypopnea Index	ODI	Mean SpO2 (%)	Nadir SpO2 (%)
Genetic characteristics	Mutation type	ms	mean± SD [n]	7.7 ± 7.35 [2]	6 ±6.93 [2]	1.7 ±0.42 [2]	8 ±7.35 [2]	97.5 ± 0.71 [2]	90 ±1.41 [2]
		ns	mean± SD [n]	4.18 ± 2.15 [4]	2.73 ± 1.27 [3]	2.10 ± 1.00 [4]	26.88 ± 42.26 [4]	71.50 ± 46.48 [4]	86.00 ± 6.00 [4]
		Standardized mean difference	Cohen's D	0.85	0.79	-0.45	-0.51	0.65	0.76
	Mutation domain	MBD	mean± SD [n]	6.57 ± 5.56 [3]	4.67 ± 5.24 [3]	1.90 ± 0.46 [3]	6.17 ± 6.09 [3]	-	90.33 ± 1.15 [3]
		TRD	mean± SD [n]	5.65 ± 0.21 [2]	3.10 ± 1.56 [2]	2.50 ± 1.27 [2]	50.65 ± 55.65 [2]	-	81.00 ± 2.83 [2]
		Standardized mean difference	Cohen's D	0.20	0.36	-0.73	-1.37	-	4.95
Clinical severity items	Epilepsy	not have	mean± SD [n]	8.10 ± 5.25 [3]	4.77 ± 5.35 [3]	3.30 ± 2.88 [3]	11.77 ± 8.34 [3]	93.67 ± 6.66 [3]	84.67 ± 9.29 [3]
		have	mean± SD [n]	4.18 ± 2.15 [4]	2.73 ± 1.27 [3]	2.10 ± 1.00 [4]	26.88 ± 42.26 [4]	71.5 ± 46.48 [4]	86.00 ± 6.00 [4]
		Standardized mean difference	Cohen's D	1.06	0.52	0.61	-0.46	0.61	-0.18
	Hand function	No hand functional abnormal-ity	mean± SD [n]	7.00 ± 8.34 [2]	-	1.55 ± 0.64 [2]	8.45 ± 6.72 [2]	96.00 ± 1.41 [2]	90.00 ± 1.41 [2]
		Non-functional handuse	mean± SD [n]	5.68 ± 2.62 [4]	-	3.23 ± 2.36 [4]	30.85 ± 40.00 [4]	69.00 ± 44.94 [4]	81.75 ± 7.18 [4]
		Standardized mean difference	Cohen's D	0.28	-	0.97	-0.64	0.69	1.32
	Sitting	No sitting abnormality	mean± SD [n]	6.50 ± 5.96 [3]	6.45 ± 6.29 [2]	2.17 ± 1.16 [3]	9.40 ± 5.03 [3]	64.67 ± 54.28 [3]	87.67 ± 4.16 [3]
		Unable	mean± SD [n]	5.73 ± 3.20 [3]	2.53 ± 1.56 [3]	3.17 ± 2.89 [3]	37.37 ± 46.32 [3]	91.33 ± 6.11 [3]	81.33 ± 8.74 [3]
		Standardized mean difference	Cohen's D	0.16	1.02	-0.45	-0.85	-0.69	0.93
	Walking	No walking abnormality	mean± SD [n]	7.00 ± 8.34 [2]	-	1.55 ± 0.64 [2]	8.45 ± 6.72 [2]	96.00 ± 1.41 [2]	90.00 ± 1.41 [2]
		Walking with support	mean± SD [n]	4.90 ± 0.85 [2]	-	2.85 ± 0.78 [2]	6.90 ± 6.22 [2]	50.50 ± 68.59 [2]	87.00 ± 5.66 [2]
		Standardized mean difference	Cohen's D	0.35	-	-1.82	0.24	0.94	0.73
		No walking abnormality	mean± SD [n]	7.00 ± 8.34 [2]	-	1.55 ± 0.64 [2]	8.45 ± 6.72 [2]	96.00 ± 1.41 [2]	90.00 ± 1.41 [2]
		Unable	mean± SD [n]	5.73 ± 3.20 [3]	-	3.17 ± 2.89 [3]	37.37 ± 46.32 [3]	91.33 ± 6.11 [3]	81.33 ± 8.74 [3]
		Standardized mean difference	Cohen's D	0.23	-	-0.68	-0.76	0.92	1.21
	Scoliosis	Had no deviation	mean± SD [n]	6.58 ± 5.26 [4]	4.77 ± 5.35 [3]	2.98 ± 2.37 [4]	9.75 ± 7.92 [4]	94.00 ± 5.48 [4]	86.25 ± 8.22 [4]
		Severe scoliosis	mean± SD [n]	5.65 ± 0.21 [2]	3.10 ± 1.56 [2]	2.50 ± 1.27 [2]	50.65 ± 55.65 [2]	46.00 ± 62.23 [2]	81.00 ± 2.23 [2]
		Standardized mean difference	Cohen's D	0.20	0.37	0.22	-1.43	1.53	0.73

AHI: apnea-hypopnea index; **MBD**: Methyl-CpG-Binding Domain; **ms**: missense mutation; **ns**: nonsense mutation; **ODI**: oxygen desaturation index; **RTT**: Rett Syndrome; **SD**: standard deviation.
